# Intelligent Bell facial paralysis assessment: a facial recognition model using improved SSD network

**DOI:** 10.1038/s41598-024-63478-x

**Published:** 2024-06-04

**Authors:** Haiping Shi, Yinqiu Fan, Yu Zhang, Xiaowei Li, Yuling Shu, Xinyuan Deng, Yating Zhang, Yunzi Zheng, Jun Yang

**Affiliations:** 1 The First Affiliated Hospital of Anhui University of Chinese Medicine, Hefei, Anhui China; 2 Anhui University of Chinese Medicine, Hefei, Anhui China

**Keywords:** SSD, CNN, Facial paralysis, VGG, Deep learning, Facial feature calculation, Diseases, Medical research

## Abstract

With the continuous progress of technology, the subject of life science plays an increasingly important role, among which the application of artificial intelligence in the medical field has attracted more and more attention. Bell facial palsy, a neurological ailment characterized by facial muscle weakness or paralysis, exerts a profound impact on patients’ facial expressions and masticatory abilities, thereby inflicting considerable distress upon their overall quality of life and mental well-being. In this study, we designed a facial attribute recognition model specifically for individuals with Bell’s facial palsy. The model utilizes an enhanced SSD network and scientific computing to perform a graded assessment of the patients’ condition. By replacing the VGG network with a more efficient backbone, we improved the model’s accuracy and significantly reduced its computational burden. The results show that the improved SSD network has an average precision of 87.9% in the classification of light, middle and severe facial palsy, and effectively performs the classification of patients with facial palsy, where scientific calculations also increase the precision of the classification. This is also one of the most significant contributions of this article, which provides intelligent means and objective data for future research on intelligent diagnosis and treatment as well as progressive rehabilitation.

## Introduction

Facial palsy, an affliction characterized by neuromuscular dysfunction of the facial region, imposes significant physical and psychological perils upon its sufferers. Individuals afflicted with this condition experience asymmetrical facial expressions, fostering feelings of embarrassment and distress in their social and interpersonal interactions. The compromised functionality of the ocular muscles can give rise to widened eye fissures and ocular dryness, severely impairing visual acuity and ocular well-being^1^. Furthermore, the engagement of lip muscles results in drooping mouth corners and limited lip mobility, thereby impeding eating, speech, and facial expressiveness, leading to challenges in daily life. The detriments caused by facial paralysis extend beyond the physical realm and exert detrimental effects on the self-esteem, self-assurance, and mental well-being of patients, thereby underscoring the pressing need for efficacious treatment options^2^.

With the progression of technology and advancements in the medical field, the repertoire of treatments available for facial palsy has expanded significantly. Clinical practice has witnessed the application of various modalities, including medication, physical therapy, and rehabilitation training, aimed at augmenting patients’ facial functionality. Among these therapeutic approaches, acupuncture, an age-old treatment modality, possesses distinctive merits. Through the stimulation of specific acupuncture points, this traditional practice harmonizes the circulation of vital energy (qi) and blood throughout the body, fostering the recovery and functional amelioration of facial muscles^3^. Acupuncture is renowned for its safety and absence of adverse effects, while also affording individualized treatment options that can be tailored to the specific circumstances of each patient. As research and implementation of acupuncture persist, its significance in the management of facial paralysis has been progressively underscored^4^. Functioning as an integrative modality within the realm of medical treatment, acupuncture engenders favorable therapeutic outcomes for individuals afflicted with facial palsy by modulating facial muscle tone, facilitating blood circulation, and enhancing nerve functionality. Clinical investigations and practical application have substantiated acupuncture’s capacity to alleviate symptoms and discomfort, enhance facial muscle functionality, and restore facial expression symmetry, thereby elevating patients’ overall quality of life^5^.

With the swift progression of artificial intelligence, its ubiquity in the domain of medicine is witnessing an exponential surge. The robust data processing capabilities and pattern recognition prowess inherent to artificial intelligence technology equip it to furnish enhanced precision and efficacy in the realm of disease diagnosis and treatment. In the realm of facial palsy research, artificial intelligence assumes a pivotal role^6^. By virtue of its capacity to analyze and process facial images, artificial intelligence technology facilitates precise identification and examination of facial attributes in patients afflicted with facial palsy. Leveraging deep learning algorithms and computer vision techniques, automated detection and analysis of facial expressions, ocular muscles, and lip muscles become feasible, thereby enabling quantitative evaluation of the patient’s facial functionality and tracking of condition alterations. This provision of crucial reference information empowers physicians and acupuncturists to craft and fine-tune treatment plans with precision^7^. Furthermore, artificial intelligence can be harnessed for the development of therapeutic assistance systems tailored to facial palsy. By erecting models and algorithms for facial attribute recognition, in conjunction with real-time facial image acquisition technology, an intelligent system can be devised to continually monitor real-time alterations in patients’ facial attributes and proffer acupuncture treatment recommendations based on these changes. Such an advancement would not only heighten the personalization and precision of treatment but also alleviate the workload of acupuncturists, thereby augmenting treatment outcomes and bolstering patient contentment.

Therefore, for the demand of intelligent treatment of acupuncture therapy combined with the current stage of artificial intelligence and deep learning technology, this paper intends to propose a facial feature study for the acupuncture treatment of Bell’s facial palsy, with the following contributions:

1. The facial palsy patients were classified into three categories: light, middle and severe according to the facial feature;
2. facial feature detection and classification of their facial palsy condition levels were achieved using improved SSD networks, with an average precision of 87.9% at three levels;
3. The refinement of the treatment time for different level patients according to the model classification results illustrates the effectiveness of acupuncture treatment for facial palsy diseases.

The rest of the investigation is organized as follows: “Related works” section introduces related works for facial feature extraction using traditional methods and deep learning methods. The related works for the facial paralysis is also given in this section. In “Establishing facial feature recognition model based on improved SSD algorithm for facial palsy patients” section, the framework for the facial paralysis classification is established; “Experiment result and analysis” section gives the experiment result and analysis; “Discussion” section is the Discussion and Conclusion is presented at the end.

## Related works

### Study on facial features of faces based on traditional features

Traditional methodologies primarily employ geometric and appearance features for feature extraction. The general procedure encompasses face detection, face feature point localization (face alignment), extraction of expression features, and subsequent classification. In terms of feature design and extraction, two main categories of features are employed: geometric features and appearance features. Geometric features encompass the shape of eyebrows, eyes, nostrils, mouth, as well as the relative positioning of feature points such as eyes and mouth. Appearance features, on the other hand, include facial furrows, wrinkles, bulges, and similar attributes. Regarding face key feature point detection and localization methods, face key point models can be categorized into 2D and 3D based on the number of feature points. 3D face feature points typically number in the thousands and find common usage in industrial settings. On the other hand, 2D face key point models typically consist of fewer than a thousand feature points. Statistical modeling methods for 2D key feature points include the active shape model method (ASM) coupled with the active appearance model approach (AAM)^8^. Furthermore, techniques based on direct regression or regression-based methods exhibit robust performance^9,10^. Prominent research endeavors concerning expression recognition subsequent to face keypoint detection encompass Matsugu’s team^11^, which explored the correlation between feature points and facial actions in a planar context to obtain expression labels. In the realm of expression recognition utilizing episodic features, Bartlett et al.^12^ devised a method incorporating Gabor features with eigenfaces to represent epigenetic attributes. Additionally, Kazemi et al.^10^ introduced a regression-based face feature point localization approach employing 68 face key feature points. Zhang et al.^13^ amalgamated appearance feature detection of facial expressions and geometric features extracted from 26 feature points, combining them with dynamic Bayesian networks for analytical purposes. Conversely, Su et al.^14^ proposed a facial expression recognition algorithm grounded in the fusion of geometric and appearance features, effectively leveraging the strengths of each modality to enhance the efficiency and performance of expression recognition feature extraction. By encompassing the geometric structural transformations of the face as well as the subtle structural disparities and alterations in local facial attributes, this approach captures a comprehensive representation of facial characteristics.

It can be seen that the traditional facial feature research is generally done by capturing the key point information in the face and completing the extraction of facial features and recognition of related information through relevant comparisons, which requires a large amount of computation and is not sufficiently adaptable. When the object has a large change, the basic features in these pictures are harder to recognize and easily fail, so a more intelligent method for facial feature extraction is urgently needed.

### Study of facial features based on deep learning methods

In recent years, deep learning techniques have experienced rapid growth and achieved remarkable advancements in image recognition technology. Convolutional neural networks (CNNs) have emerged as dominant models, with notable examples such as GoogleNet, AlexNet, ResNet, and VGGNet^15,16^. The progress in computing technology and the development of hardware devices like GPUs have provided substantial support for the parameter calculations involved in training deep CNN models. Additionally, the era of big data, the proliferation of research institutions and researchers in expression recognition-related fields, and the availability of abundant expression databases have collectively propelled the swift advancement of expression recognition, both in terms of software and hardware. For instance, Mollanosscini et al.^17^ proposed a modified GoogleNet model-based single component network architecture that achieved improved results in facial expression recognition. Cesar Isaza introduced a dynamic setpoint model for detecting face parts and determining fatigue status^18^. Huynh et al. designed a 3D CNN with a gradient enhancement algorithm to extract key features from video images for human fatigue state determination; however, the DDD dataset poses considerable challenges and requires substantial computational resources^19^. Deep learning techniques for facial feature extraction can also be applied in assessing disorders such as depression. Zhu et al.^20^ proposed a dual-stream CNN network that captures facial appearance features and facial motion features, where one network processes the original image while the other network takes input from extracted optical flow features. Subsequently, two fully connected layers perform feature dynamic history histogram (FDHH) analysis on the visual feature space to model temporal features. Jan et al.^21^ utilized the VGG architecture to extract visual features from face images using FDHH, and applied it to model temporal features. Zhou et al.^22^ employed a CNN with a global average pooling (GAP) layer to establish correspondence between different facial regions and recognize depression from facial images. They combined the responses of different facial regions to obtain the final recognition results. Moreover, the trained depth model was combined with a Depression Activation Map (DAM) to visualize the depression level across different facial regions. Chowdary et al.^23^ dals with emotion recognition by using transfer learning approaches with pre-trained networks of Resnet50, vgg19, Inception V3, and Mobile Net. The result shows that the emotion detection accuracy could reach more than 96%. Mukhiddinov et al.^24^ proposed approach employs the AffectNet image dataset, which includes eight types of facial expressions with different light intensity.

### Assessment and analysis of facial paralysis

If facial paralysis patients receive early treatment, they can fully recover from facial paralysis; If left untreated, there may be sequelae. The recovery period is the most important period for facial nerve repair, during which facial muscle training can maximize the recovery of facial nerve function^25^. The traditional method of facial muscle training mainly involves patients undergoing mirror feedback training, which guides them to repeatedly perform facial movements such as raising eyebrows, frowning, closing eyes, showing teeth, and pouting when facing the mirror. This training process is dull and tedious, and due to the need for repeated training, the patient’s enthusiasm is also not high. In addition, due to significant differences in the degree of facial paralysis, the differences between different patients have not been quantified, and personalized training plans cannot be customized for patients, which cannot achieve ideal rehabilitation effects. The commonly used evaluation scales in clinical practice include the House Brackmann Facial Nerve Function Grading Scale (HBGS), Facial Nerve Evaluation System 2.0 (FNGS 2.0), Burres Fisch Facial Nerve Scoring System, Nottingham System, Facial Disability Index (FDI), etc.^26^. This type of evaluation method based on scales and clinical observations places high demands on the clinical experience of doctors. With the development of artificial intelligence technology, people in the field of facial paralysis research are gradually using more detailed network methods based on human multi-source signal features to study the facial features of facial paralysis patients. Barbosa et al. extracted facial feature points based on human iris segmentation and mixed classifiers for facial nerve function assessment^27^. Wang et al. quantified the asymmetry of facial nerve function in both static and dynamic conditions, and evaluated facial nerve function by combining the two results. However, this method may lead to incorrect quantification results due to misclassification^28^. Yoshihara et al. used the AAM model for initial facial feature point detection, and then used image blocks centered around the initial feature points as inputs to improve the feature points of the deep convolutional neural network. However, they did not quantitatively evaluate facial nerve function^29^ Dittmar et al. attempted to quantitatively describe facial nerve function using static extraction algorithms based on 3D features^30^. Jiang et al. developed a facial nerve function evaluation cloud platform based on machine learning and manual input from doctors, which can output Sunnybrook facial paralysis grading system scores, but did not provide the accuracy of the experimental method^31^.

Deep learning effectively avoids human intervention and directly performs end-to-end feature learning. Applying deep learning techniques to facial expression recognition can more easily and effectively obtain facial-related features, thus helping researchers to accomplish related tasks. Therefore, it is important for the diagnosis and treatment of facial palsy to use deep learning technology to optimize its features in a hierarchical manner, which is an important indicator for the progressive demonstration of treatment effects. In the traditional treatment of facial paralysis, it is believed that observing the details of facial features is difficult to capture, and deep technology based facial expression research has achieved good results in the field of micro expression recognition. This indicates that deep learning techniques can effectively capture facial details and are of great significance for the rehabilitation of patients with facial paralysis.

## Establishing facial feature recognition model based on improved SSD algorithm for facial palsy patients

### Facial palsy model classification and data creation

Bell's facial palsy is an acute ipsilateral facial nerve paralysis with an unknown cause. It is often attributed to facial nerve edema or demyelination resulting from viral infection or exposure to cold temperatures. Clinical manifestations primarily include incomplete eyelid closure (difficulty in raising and closing the eyebrows, inability to frown) and distorted corners of the mouth (bone and cheek leakage). Bell Facial Paralysis is usually unilateral, causing sudden weakening of facial muscles on one side. In most cases, this weakness is temporary and significantly improves within a few weeks. People with Bell's palsy may experience sagging on one side of the face, a unilateral smile, and difficulty in closing the affected eye. The appropriate treatment is crucial for reducing long-term complications, while improper treatment can lead to facial muscle spasms and exacerbate complications such as crocodile tears^32^. To grade the facial features of individuals undergoing acupuncture treatment for Bell's facial palsy, different levels are assigned based on the severity of the condition and the observed features. In this study, the condition of facial palsy patients was classified into three categories: light, middle, and severe, following recommendations and instructions provided by medical experts. Table 1 presents the main features associated with each level.

**Table1:** The clinical feature level of the facial paralysis.

Level	Clinical feature
Light	Slight facial muscle weakness or paralysis with asymmetrical but milder expressions Eyelid weakness, but can be largely closed Slight drooping of the lips, but it does not significantly affect the lip movements and expressions Facial skin may be slightly lax on the affected side with reduced folds
Middle	Moderate facial muscle weakness or paralysis with marked asymmetry of facial expression Weakness of the eyelid muscles, resulting in a significant widening of the eye fissure and possible dryness and discomfort in the eyes Significant drooping of the lips, limited lifting of the corners of the mouth, and some impairment of mouth and lip movements and expression Visible facial skin laxity and reduction of folds
Severe	Severe facial muscle weakness or paralysis with highly asymmetrical expressions Weakness of the eyelid muscles, marked widening of the eye fissure, and limited eye rotation Severe drooping of the lips, difficulty in lifting the corners of the mouth, affecting mouth and lip movements and expressions Severe facial skin laxity with significant reduction or loss of folds

Through the division of facial palsy disease features under different levels, we can see that the changes of expressions and facial features are more obvious, so the extraction of relevant features can be well done by deep learning methods, which completes the classification of relevant levels, thus realizing the later classification analysis and personalized treatment design.

### A grading evaluation model for patients with facial palsy based on improved SSD patients

**Figure 1:**
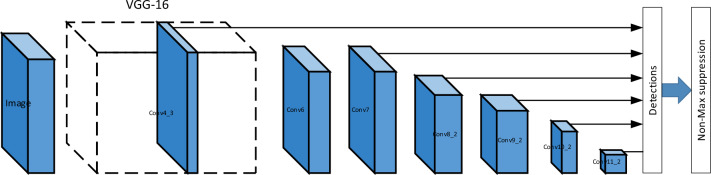
The structure of the SSD.

SSD (Single Shot MultiBox Detector) is an algorithm for target detection with a network structure and features that make it advantageous in terms of real-time and accuracy. The SSD algorithm uses a convolutional neural network (CNN) based multi-scale feature extraction strategy that combines different levels of feature maps to detect targets^33^ The network structure of SSD consists of two parts: the base network and the multiscale feature layer. The base network usually uses some popular convolutional neural networks, such as VGG16 or ResNet, for extracting high-level semantic features of images. The multi-scale feature layer, on the other hand, is used to detect targets of different sizes. The structure of the network is shown in Fig. 1.

The features of SSD have three main aspects, firstly, it has multi-scale feature fusion, SSD introduces multiple feature layers in the network, which have different scales and semantic information. By fusing these feature layers, SSD can detect targets at different scales, thus improving the accuracy and robustness of detection. Secondly, multi-scale anchor frames can be implemented. SSD performs target detection by placing anchor frames of different scales and aspect ratios on each feature layer. These anchor frames can cover targets of different sizes and shapes, enabling SSD to effectively detect targets of multiple scales. Finally the network is able to decode the detection results and the SSD performs classification and bounding box regression on each anchor box by convolutional and prediction layers^34^. The classification layer is used to determine the presence of targets in the anchor frame and classify them into different categories, and the bounding box regression layer is used to adjust the position and size of the anchor frame to fit the targets more accurately. For the above three features, the SSD network can be divided into three parts, i.e., multi-scale anchor frame generation, bounding box regression, and loss function training, where multi-scale anchor frame generation means that given a feature layer, and its corresponding input image size and stride (stride), the position and size of the anchor frame can be calculated by the following equation:

1 $$box_{width} = base_{width} *sqrt(aspect_{ratio} )$$

2 $$box_{height} = base_{height} /sqrt(aspect_{ratio} )$$

3 $$center_{x} = (i + 0.5)*stride$$

4 $$center_{y} = (j + 0.5)*stride$$

5 $$anchorbox = [center_{x} ,center_{y} ,box_{width} ,box_{height} ]$$

where $i$ and $j$ represent the position indexes on the feature map, respectively,${\text{base}}_{{{\text{width}}}}$ and ${\text{base}}_{{{\text{height}}}}$ are the width and height of the reference anchor frame, and ${\text{aspect}}_{{{\text{ratio}}}}$ represents the aspect ratio. Then the bounding box regression is performed

6 $$predictedbox_{x} = offset_{x} *anchorbox_{width} + anchorbox_{center\;x}$$

7 $$predictedbox_{y} = offset_{y} *anchorbox_{height} + anchorbox_{center\;y}$$

8 $$predictedbx_{width} = exp(offset_{width} )*anchorbox_{width}$$

9 $$predictedbox_{height} = exp(offset_{height} )*anchorbox_{height}$$

where,${\text{offset}}_{{\text{x}}}$, ${\text{offset}}_{{\text{y}}}$, ${\text{offset}}_{{{\text{width}}}}$ and ${\text{offset}}_{{{\text{height}}}}$ are the bounding box offsets predicted by the network, and ${\text{anchorbox}}_{{{\text{center}}\;{\text{x}}}}$, ${\text{anchorbox}}_{{{\text{center}}\;{\text{y}}}}$, ${\text{anchorbox}}_{{{\text{width}}}}$ and ${\text{anchorbox}}_{{{\text{height}}}}$ are the center coordinates and width and height of the anchor box.

SSD uses a multi-task loss function that combines classification loss and bounding box regression loss to train the network. The classification loss uses the cross-entropy loss as shown in Eq. (10), and the bounding box regression loss (11) uses the smoothed L1 loss (12) to complete the total loss calculation.

10 $$L_{c} ls = - \, \sum {(y_{t} rue_{c} ls*log(y_{p} red_{c} ls) + (1 - y_{t} rue_{c} ls)*log(1 - y_{p} red_{c} ls))}$$

11 $$L_{r} eg = \, \sum {(y_{t} rue_{r} eg*SmoothL1(y_{p} red_{r} eg - y_{t} rue_{r} eg))}$$

12 $$L = \, \alpha L_{c} ls + \, \beta L_{r} eg$$

**Figure 2:**
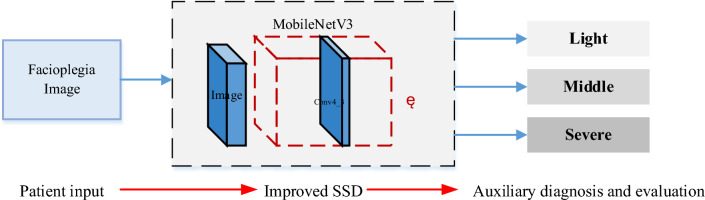
The framework for the facial paralysis classification.

The VGG network is enhanced by modifying the SSD (Single Shot MultiBox Detector) structure, wherein multiple layers, including the final pooling layer, are extracted as the feature layer. Default detection boxes of various scales are utilized to detect targets of different sizes. Each feature layer has a predefined number and size of target frames. The target class is determined by propagating the data of each default detection frame through the fully connected layer, and the best detection candidates are selected based on target accuracy and the overlap rate of rectangular frames. In this study, the VGG network is further improved by incorporating the MobileNetV3 network. The computational workload of the neural network primarily lies in the feature extraction phase, particularly in the VGG network. However, MobileNetV3 reduces the computational requirements during feature extraction, leading to a reduction in model size and increased speed. As a result, MobileNetV3 is well-suited for mobile devices, offering advantages in terms of time overhead and model size. It has been demonstrated that reducing the number of systematic feature extractions significantly reduces both the time and computational overhead^35^. Leveraging the excellent performance of MobileNetV3 on mobile devices, this paper replaces the VGG backbone network of SSD with MobileNetV3. A pooling layer operation follows, reducing the size of feature maps generated by the preceding layers of the network in a layered manner. This replacement of the VGG-16 component, as depicted in Fig. 1, effectively reduces model complexity, enhancing its applicability. The algorithm framework obtained by improving the SSD network is illustrated in Fig. 2.

## Experiment result and analysis

In this study, the Facial Paralysis Image Database (FPID)^36^, a publicly available facial paralysis image dataset, was used. The database was released to serve as a resource for facial palsy education and research. Initially, to demonstrate the utility of the database the relationship between the level of facial function and the perceived emotion expression was successfully characterized using a machine learning-based algorithm. The database is composed of 480 high-resolution images from 60 participants, 10 healthy subjects, and 50 patients with different paralysis level. Based on the classification level of facial paralysis disease as described previously and the improved SSD facial feature recognition algorithm, the experimental data of facial paralysis patient images in this dataset are collected and analyzed.

### The model training and classification result

**Figure 3:**
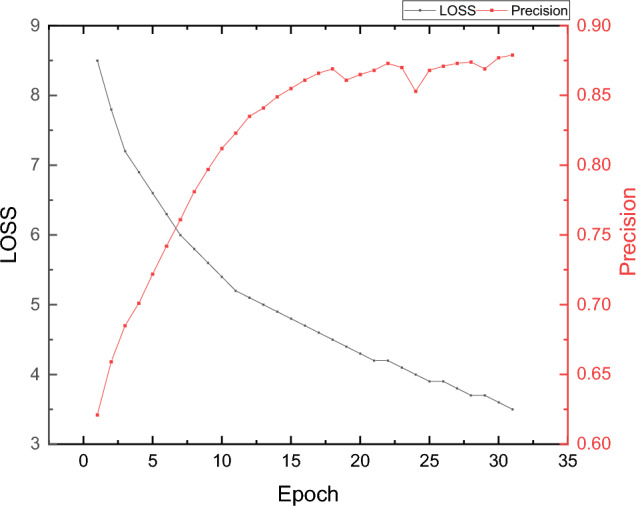
The training loss and precision of the proposed model.

The model training is performed using the facial paralysis classification framework shown in Fig. 2, and the variation of loss function and model precision throughout the training process is shown in Fig. 3.

**Figure 4:**
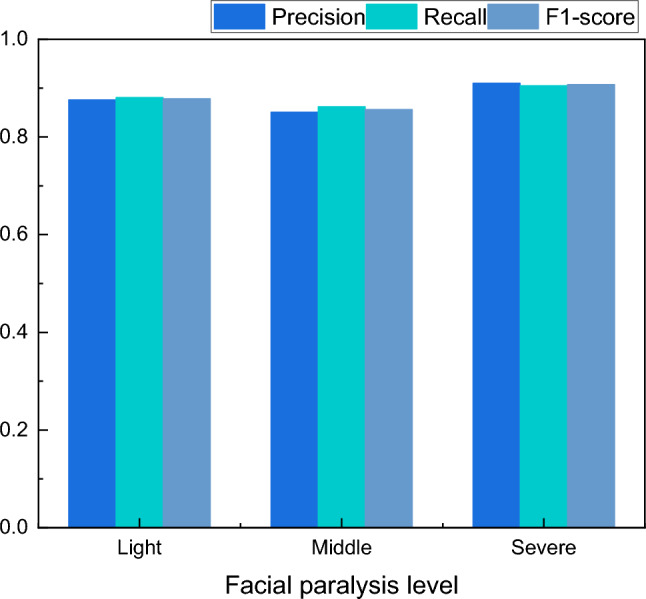
The classification result for the facial paralysis level.

Figure 3 illustrates the gradual change of the loss function and model precision as the number of iterations increases. It can be observed that the model's precision fluctuates after a certain number of training iterations. However, the final precision remains relatively stable, reaching an average precision of 87.9%. The detailed results of the classification of different facial palsy classes were compared and analyzed using metrics such as Precision, Recall, and F1-score. These results are presented in Table2 and Fig. 4.

**Table 2:** The classification result for the facial paralysis level.

Level	Precision	Recall	F1-score
Light	0.876	0.881	0.878
Middle	0.851	0.862	0.856
Severe	0.91	0.905	0.907

According to the recognition effect of the three categories of facial palsy patients' classification shown in Fig. 4, it can be seen that the recognition precision for the middle category and the highest recognition precision for the sever is due to the fact that the middle category also has certain deviations in the actual classification process, which is often carried out by the exclusion method, so its recognition effect may be poor.

### Methods comparison for the facial feature extraction

**Figure 5:**
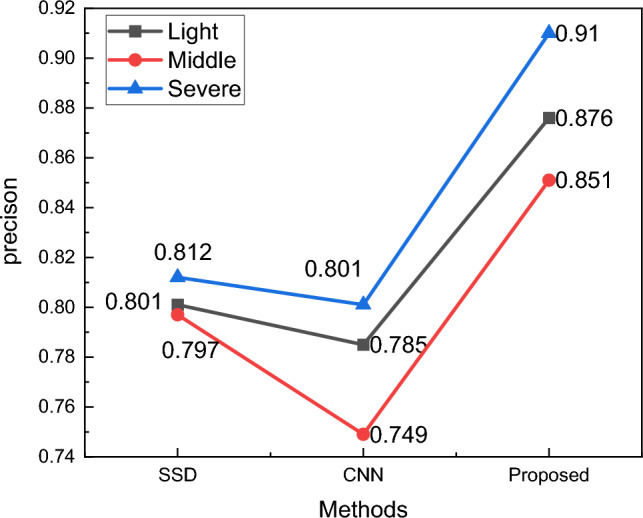
The facial paralysis level classification using different methods.

After the training of the model and the analysis of the results were completed, method comparison experiments were conducted to better illustrate the usefulness of the improved SSD network. In the comparison, the unimproved SSD and the traditional CNN method were selected, whose CNN building model and structure are similar to the SSD network structure, and the specific results obtained are shown in Fig. 5.

According to the data in Fig. 5, it can be seen that the proposed method has the highest recognition rate for patients with all three degrees of facial palsy, and according to the curve changes, it is also easy to see that the three methods have the highest recognition rate for severe for Light, middle and severe conditions, and further enhancement of the balance and generalization line of the model is needed in future research.

### The classification analysis among patients with different levels

**Figure 6:**
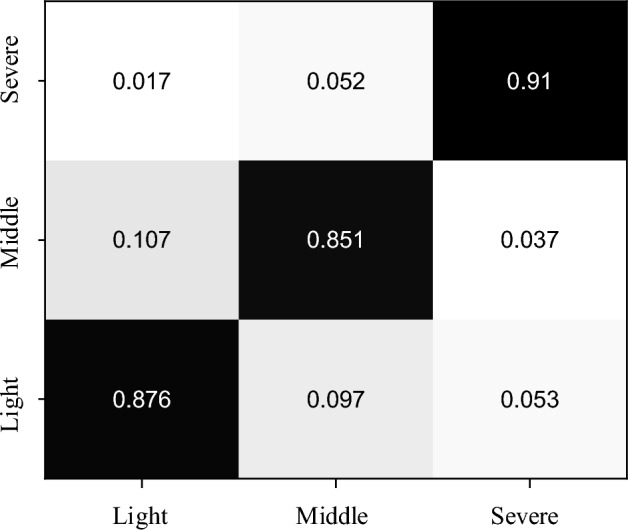
The confusion matrix for the different levels.

After completing the training of the model and the construction of the framework, we further analyzed the rows according to the classification results, and for the data used, the overall confusion matrix is shown in Fig. 6.

**Figure 7:**
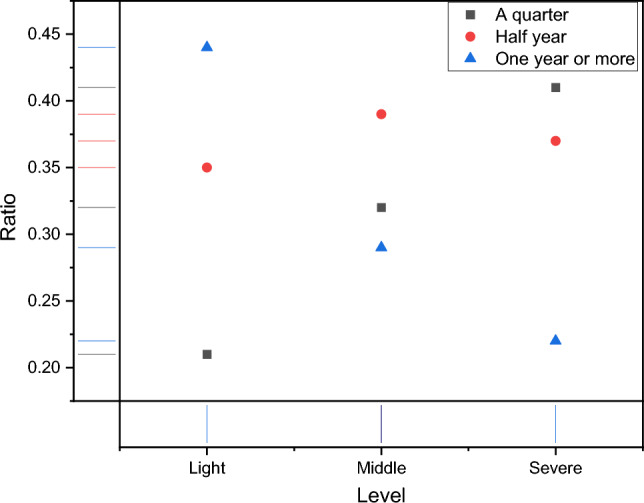
The ration distribution of treatment time for patients with facial paralysis.

In Fig. 6, it can be seen that there are more misclassification results for the MIDDLE class data, which requires further research on how to level out the model performance in future studies. After completing the analysis related to the model data, we conducted statistics on the treatment years of the data used, and the results are shown in Fig. 7.

We conducted statistical analysis based on the treatment data of relevant patients in the database used. They were all treated using the standard treatment plan of the hospital where the dataset creator was located, and the degree of facial paralysis was alleviated. In Fig. 7, we can see that as the treatment time increases, the severity level of patients gradually decreases, and for patients who only receive one quarter of treatment, the proportion of light is found to be the lowest. The results in Fig. 7 can prove to some extent that the acupuncture and moxibustion method changes the facial features of patients and its positive clinical treatment effect.

## Discussion

Facial palsy is a neurological disorder that profoundly impacts the facial expression muscles and significantly affects patients' quality of life and social interactions. While acupuncture has been traditionally used as a treatment for facial palsy, its effectiveness is challenged by the complexity of the condition. Fortunately, the rapid progress of artificial intelligence technology offers new possibilities for facial palsy research and treatment. In this study, we focused on leveraging an improved SSD network for facial feature recognition in patients with Bell's facial palsy. By enhancing the conventional CNN and SSD models and incorporating the efficient MobileNetV3 architecture, our improved SSD network demonstrated notable advantages in facial feature recognition. Firstly, the improved SSD exhibited superior performance in terms of recognition rate. The integration of MobileNetV3 into the network structure enhanced the accuracy of the model, enabling more precise identification of facial features associated with facial palsy. Secondly, the improved SSD is superior in speed. It achieved a balance between recognition speed and accuracy, which is crucial for practical clinical applications. Acupuncturists can benefit from fast and accurate recognition of facial features, as it aids in assessing patients' conditions and monitoring treatment progress effectively. After improving the model, its running time has been reduced by more than 20% compared to before. By integrating MobileNetV3, the improved SSD network structure is lighter, reducing computational burden and improving processing speed. The design of MobileNetV3 focuses on improving performance on mobile and embedded devices, further accelerating recognition speed by optimizing convolution operations. The improved SSD adopts an efficient network architecture, effectively balancing accuracy and computing speed, making it perform better in real-time applications. This is crucial for facial feature recognition, especially in clinical applications. Rapid and accurate recognition of facial features can help acupuncture and moxibustion better assess patients' conditions and treatment progress. Overall, the improved SSD network presented in this study offers promising advancements in facial feature recognition for patients with facial palsy. Its enhanced accuracy and speed make it a valuable tool in clinical settings, facilitating improved patient assessment and treatment monitoring.

The application of artificial intelligence in the future treatment of facial palsy has a broad prospect and great potential. First, AI can provide more accurate and efficient support for the diagnosis and treatment of facial palsy. By using big data and machine learning algorithms, more accurate facial feature recognition models can be established to assist doctors and acupuncturists to make more accurate diagnosis and develop personalized treatment plans. Secondly, AI can provide real-time monitoring and feedback during the treatment of facial palsy. By using intelligent facial image acquisition devices and AI algorithms, it can track the changes of patients' facial features in real time, evaluate the treatment effect and adjust the treatment plan in time. This will greatly improve the accuracy and personalization of treatment, while reducing the workload of the acupuncturist. In addition, AI can also promote the development of intelligent and personalized facial palsy treatment. By combining AI with technologies such as virtual reality and augmented reality, intelligent systems for facial palsy rehabilitation training can be developed. These systems can provide personalized rehabilitation training plans based on the patient's specific situation and guide the patient through training with real-time monitoring and feedback. This can not only increase patient participation and motivation, but also improve the rehabilitation effect and accelerate the rehabilitation process. Through continuous exploration and innovation, artificial intelligence will bring more accurate, personalized and effective solutions for facial palsy treatment.

## Conclusion

This study is based on the improved SSD algorithm for facial feature recognition of Bell's facial palsy acupuncture treatment patients, so as to realize the intelligent grading evaluation of facial palsy patients' conditions. In this paper, the grading of light, middle and severe patients is completed based on the existing facial palsy data, and the model recognition accuracy and computational efficiency are improved by improving the existing SSD network. The experimental results show that by introducing MobileNetV3 to replace VGG, the prediction accuracy of the model can be greatly improved, and the average recognition rate of the three categories of patients reaches 87.9%, which is higher than 80.3% of the unimproved method. Meanwhile, this paper analyzes the application prospects of AI methods within this field, illustrates the usability of deep learning methods, and provides new ideas for the future development of smart medicine.

However, there are also some limitations of the study. The research was conducted with a small dataset and focused on only three levels of classification. To further enhance the reliability and generalizability of the model, future research should expand the dataset, ensure data privacy and security, and improve the robustness of the algorithm.

## Data Availability

If anyone needs a dataset used in the article, they can contact the corresponding author on reasonable request.
